# Assessment of Fecal Exposure Pathways in Low-Income Urban Neighborhoods in Accra, Ghana: Rationale, Design, Methods, and Key Findings of the SaniPath Study

**DOI:** 10.4269/ajtmh.16-0508

**Published:** 2017-07-17

**Authors:** Katharine Robb, Clair Null, Peter Teunis, Habib Yakubu, George Armah, Christine L. Moe

**Affiliations:** 1 Center for Global Safe Water, Sanitation, and Hygiene, Hubert Department of Global Health, Rollins School of Public Health, Emory University, Atlanta, Georgia;; 2 Mathematica Policy Research, Washington, District of Columbia;; 3 Centre for Zoonoses and Environmental Microbiology, Centre for Infectious Disease Control, RIVM, Amsterdam, The Netherlands;; 4 The Noguchi Memorial Institute for Medical Research of the University of Ghana, Accra, Ghana

## Abstract

Rapid urbanization has contributed to an urban sanitation crisis in low-income countries. Residents in low-income, urban neighborhoods often have poor sanitation infrastructure and services and may experience frequent exposure to fecal contamination through a range of pathways. There are little data to prioritize strategies to decrease exposure to fecal contamination in these complex and highly contaminated environments, and public health priorities are rarely considered when planning urban sanitation investments. The SaniPath Study addresses this need by characterizing pathways of exposure to fecal contamination. Over a 16 month period, an in-depth, interdisciplinary exposure assessment was conducted in both public and private domains of four neighborhoods in Accra, Ghana. Microbiological analyses of environmental samples and behavioral data collection techniques were used to quantify fecal contamination in the environment and characterize the behaviors of adults and children associated with exposure to fecal contamination. Environmental samples (n = 1,855) were collected and analyzed for fecal indicators and enteric pathogens. A household survey with 800 respondents and over 500 hours of structured observation of young children were conducted. Approximately 25% of environmental samples were collected in conjunction with structured observations (n = 441 samples). The results of the study highlight widespread and often high levels of fecal contamination in both public and private domains and the food supply. The dominant fecal exposure pathway for young children in the household was through consumption of uncooked produce. The SaniPath Study provides critical information on exposure to fecal contamination in low-income, urban environments and ultimately can inform investments and policies to reduce these public health risks.

## INTRODUCTION AND RATIONALE

### Urbanization, sanitation, and public health in low-income countries.

Rapid urbanization has contributed to a growing sanitation crisis in cities in low-income countries. Over half of the global population now resides in cities, and this proportion continues to grow.^1–3^ Globally, it is estimated that one-third of the world’s urban population lives in slums, and in sub-Saharan Africa, close to two-thirds of the urban population lives in slums.^4^ Slums in sub-Saharan Africa are growing at a rate of 4.5% per year, double the global average.^5^ Rapid population growth has outpaced the capacity of existing urban infrastructure, including water and sanitation systems, to provide basic services. As a result, urban dwellers in low-income settlements may live in very polluted environments and face elevated risks of enteric disease due high population density, communal exposures, and inadequate infrastructure and services to safely contain and dispose of excreta and solid waste.^6–10^ In rapidly growing urban informal settlements, under-five morbidity and mortality rates can be higher than in rural areas.^11–14^

Globally, an estimated 2.5 billion people lack access to safe sanitation, and the Millennium Development Goal (MDG) for sanitation was not achieved.^15^ Goal Six of the 2015 Sustainable Development Goals (SDGs) moves beyond the MDG of increasing access to toilets and now includes safe fecal sludge management (e.g., the management of excreta from containment to emptying, transport, treatment, and safe disposal or reuse) which is especially critical in densely populated areas.^16^ Goal Eleven of the SDGs also relates to urban sanitation and includes targets to make cities safer, more resilient, and sustainable.^17^ The SDGs place special emphasis on reducing inequality and reaching the poor. Recently fecal waste flow diagrams have been developed for many low-income cities to describe the transport and fate of fecal sludge through the city. These diagrams have been used for sanitation advocacy and show that the majority of fecal sludge often remains within the residential environment and is untreated.^18^ The vast majority of cities in low-income countries lack the infrastructure, capacity, or financial systems to deliver fecal sludge management services.^18^ To meet the SDGs, governments and development partners need direction on how limited funding can best be used to improve sanitation and fecal sludge management and reduce public health risks from exposure to fecal contamination in the urban environment.

Addressing the water and sanitation needs of low-income urban populations poses complex challenges. Improvements in sanitation infrastructure are difficult because of rapid growth, lack of space, and the illegal status of many slums.^19^ Uncertain housing tenure impedes residents’ ability and motivation to make sanitation infrastructure improvements.^20,21^ Many governments in low-income countries have scarce resources to provide adequate water and sanitation services to growing populations.^22^ Urban sanitation investments are often driven by engineering and drainage considerations and tend to prioritize business districts rather than public health needs.^7,23^

There are relatively few descriptions in the scientific literature of sanitation interventions that reduced the enteric disease burden in urban settings.^19^ Lack of progress in this area may stem in part from inadequate information about the relative public health importance of specific exposure pathways to fecal contamination (i.e., routes such as drinking water, open drains, floodwaters, etc.). In urban settings with complex, interrelated exposure pathways, sanitation interventions at the household level may do little to reduce overall exposure to fecal contamination, and subsequent risk of enteric disease, if exposure is not also reduced in the public domain.^24,25^ For example, a study of a piped sewerage intervention in a city in Brazil reported a 22% reduction of diarrhea prevalence in children under 3 years of age. The investigators noted that the presence of an indoor toilet was not associated with reduction in diarrhea, but neighborhood coverage with sewerage was associated with a reduction in diarrhea.^26^ The study emphasized the need for sanitation interventions in the public domain to reduce exposure to fecal contamination both in and outside the household environment. Public domain interventions are especially important in densely populated urban environments where the division between public and private space is less distinct. In many low-income, urban environments, families use public spaces for traditionally domestic (private domain) activities such as food preparation, and the private domain is frequently used for public purposes, such as the operation of small businesses.^27^ The abundant sources of fecal contamination within the public domain, combined with a wide range of exposure behaviors and high population density, place urban residents, especially children, at a high risk of exposure to fecal contamination.^28^

### The SaniPath Study.

This study focuses on exposure assessment and was designed to characterize risks from fecal contamination in low-income, urban environments and identify the dominant fecal exposure pathways. The interdisciplinary approach presented in this study is novel in a number of ways: 1) The data collection strategy was based on requirements for quantitative microbial risk assessment (QMRA); 2) The study included a comprehensive set of substudies of a wide range of exposures in both the public and private domains; and 3) The breadth of data, both microbial and behavioral, generated from a single setting is unmatched. This article describes the rationale, design, study sites, methods, and analytical approach of the SaniPath Study, and highlights and synthesizes key findings.

### Approaches to estimating risks from exposure to fecal contamination.

Shuval and others proposed the theory that, at the lower end of the socioeconomic spectrum, there is a threshold below which investments in water and sanitation alone result in little detectable improvement in health outcomes.^29^ The authors suggested that under conditions of multiple and simultaneous routes of disease transmission, and where levels of nutrition and personal hygiene are low, reducing exposure by targeting a nondominant pathway (e.g., improving the public water supply) would not necessarily result in improved health outcomes. This theory is consistent with numerous reports of water, sanitation, and hygiene (WASH) interventions that have failed to demonstrate improved health outcomes.^30,31^ Understanding which pathway(s) are the dominant routes of exposure can help decision-makers plan interventions that are more likely to maximize the public health benefit.

**Figure 1.**
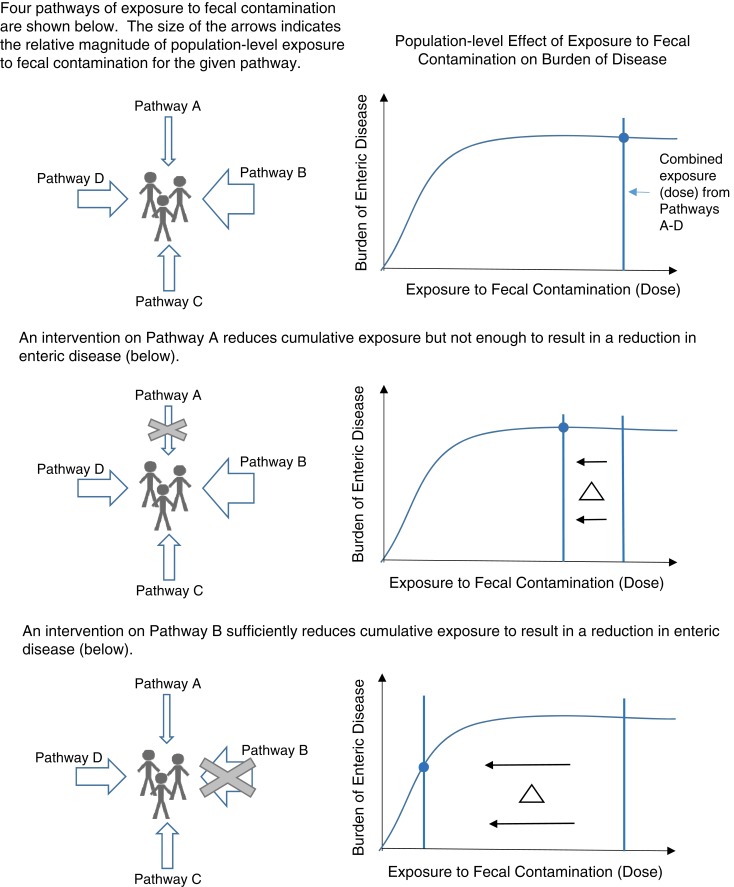
Maximizing public health impact by targeting sanitation interventions toward dominant fecal exposure pathways. This figure appears in color at www.ajtmh.org.

Dominant exposure pathways may be identified through epidemiological studies that examine the effects of intervention(s) on health outcomes. Briscoe demonstrated that due to nonlinear dose-response curves, if diarrheal disease incidence falls sharply after a WASH intervention, the affected route can be considered the primary route of transmission. However, if the disease incidence does not fall sharply, no conclusions can be drawn about the relative importance of different exposure routes.^32^ Additionally, Briscoe explained how reducing transmission from a known dominant transmission route may be necessary but not sufficient to reduce disease incidence if there are significant secondary transmission routes. Alternative approaches are needed to assess the relative importance of fecal exposure routes in complex, highly contaminated environments. Figure 1 illustrates how public health impact may be maximized by targeting WASH interventions toward dominant fecal exposure pathways.

Epidemiological studies of sanitation and health often use incidence of diarrheal disease or anthropometric indicators as an outcome and subsequently seek to characterize exposures. Health outcome data on enteric diseases are challenging to collect and interpret. For example, studies typically rely on self-report or clinical records to measure diarrheal disease incidence. Self-reported diarrhea is subject to biases, and clinical data underestimates the true burden of enteric infection. Diarrhea surveillance is costly, and without stool samples, enteric infection cannot be confirmed or linked to specific pathogens. Furthermore, even in the absence of symptoms, enteric infection has been shown to be detrimental to child growth and development.^33^

Instead of attempting to measure health outcomes directly this study aims to quantify exposure, to fecal contamination as a proxy for health effects. QMRA uses data on pathogen concentrations and human behaviors related to intake of fecal contamination to estimate exposure risk. Compared with epidemiological studies, QMRA is better suited to examine low levels of risk and health effects that are difficult to measure.^34^ Focusing on exposure recognizes the fundamental concept that health effects are conditional on exposure and without exposure there would be no health effects. However, extrapolating from exposure to health effects may overestimate the risk of health effects because 1) due to differences in immunity, not all who are exposed will develop health effects; 2) some acute health effects will be too mild to be measurable; and 3) other, long-term health effects will be delayed in onset, so that they may not be measured during the timeframe of an epidemiological study. Conversely, epidemiological studies that focus on the relation between interventions and population health tend to underestimate risk, because of under-ascertainment and underreporting of health outcomes. The true burden of health effects may be bounded by the QMRA estimates of exposure and epidemiologic estimates of health effects. This gap between risk assessment and epidemiology becomes even more relevant as additional health outcomes associated with fecal exposure (e.g., environmental enteropathy, stunting, and cognitive deficits) are being recognized.^35,36^ In this context, not only short-term exposure may be relevant, but cumulative exposure over extended periods is also important, similar to considering the risks posed by chronic exposure to toxic substances or ionizing radiation.

The World Health Organization (WHO) uses a QMRA framework to assess sanitation-related public health risks from wastewater irrigation and as the evidence base for the WHO Water Safety Planning and Sanitation Safety Planning methodologies.^37–39^ We suggest that fecal exposure pathways could be ranked to provide guidance on where to target WASH interventions, which may lead to reduction of adverse health outcomes.

QMRA studies often focus on a single environmental exposure pathway (e.g., drinking water) and estimate the health risk attributable to that pathway (for a target pathogen). The majority of QMRA studies in low- and middle-income countries have focused on enteric disease risk from drinking water or drainage channels.^40–45^ In low-income, urban environments with poor sanitation, human and animal excreta contaminate multiple environmental compartments simultaneously, and the combined contamination in these compartments (drinking water, foods, contact with surfaces, bathing water, etc.) contributes to total fecal exposure. As the health risks associated with exposure depend on the magnitude of exposure (the dose), it is essential to obtain quantitative information about the concentrations of fecal microbes in all the relevant environmental compartments, as well as the duration, frequency, and type (contact or ingestion) of exposure.

Few exposure assessments have objectively quantified exposure behavior and fecal contamination associated with multiple pathways in low-income urban environments. Previous studies have relied on perceived risks and expert opinion regarding environmental health hazards.^46–49^ Given the numerous and varied exposure routes, assessing fecal contamination in this manner is problematic. A 2010 QMRA study by Labite and others collected environmental samples to assess multiple sources of exposure to fecal contamination in Accra, Ghana, but the sample collection and microbiological analyses used to quantify environmental contamination were limited.^50^ Another QMRA study by Katukiza and others analyzed environmental samples for a variety of enteric pathogens to assess pathways of exposure to fecal contamination in densely populated settlements in Kampala, Uganda.^51^ Both studies used assumptions about the frequency and type of contact with fecal contamination. The studies used point estimates of intake volumes and frequency of exposure, rather than distributions, without information about uncertainty of the estimates. Additionally, the studies assumed each pathway was independent and there was no interaction between exposure pathways and hygiene behaviors.

Comprehensive data on multiple exposure routes in complex, low-income urban environments are needed for quantitative exposure assessment and to identify the fecal exposure pathways that pose the greatest public health risk. To address this need, we conducted an in-depth, interdisciplinary research study to characterize pathways of exposure to fecal contamination in four low-income urban neighborhoods in Accra, Ghana. We measured concentrations of fecal microbes in the environment and characterized the behavior of adults and children that were associated with exposure to fecal contamination. The goal of the SaniPath Study was to provide public health evidence to inform WASH intervention strategies, investments, and policies to more effectively target the critical exposure pathways.

## METHODS

### Study design.

Fecal exposure pathways in both public and private domains were assessed in eight substudies of the public domain: beaches, public toilets, schools, nurseries, open drains, markets, urban agricultural areas, and flood zones. Fecal exposure pathways within the private household domain were also examined. Each substudy focused on a specific setting and the fecal exposure pathways relevant to that setting. This approach allowed for comparison of risk of exposure to fecal contamination within and across exposure pathways. The substudies and relevant exposure pathways were identified through literature review and pilot fieldwork and were vetted with members of a local advisory board.

Most of the substudies focused on public domain exposures because 1) this is the area that is most amenable to public sanitation investment, and 2) in crowded, low-income areas, the majority of exposure behavior occurs in communal space. However, the private domain (household) is where children under 5 years of age, the group most vulnerable to, and impacted by, the health risks of poor sanitation, spend the majority of their time. Therefore, the household substudy included detailed data collection on child behavior and more extensive environmental sampling.

### Study site: Accra, Ghana.

The SaniPath Study was conducted over a 16-month period from June 2011 to December 2012. Accra, the capital city, had an estimated population of over 2.1 million in 2010 and a growth rate of 3.1% per year.^52,53^ In 2011, it was estimated that 38.4% of the population of Accra lived in slums.^54^ Common to many rapidly growing cities in sub-Saharan Africa, basic water and sanitation services have not matched growth. Within the Greater Accra Metropolitan Area (GAMA), 15% of residents are served by a sewerage system, 41% rely on fee-for-use public toilets, 33% have on-site facilities, 3% use bucket or pan latrines, and 7% do not have access to any improved sanitation facility.^55^ In GAMA, 64% of households have access to piped water,^53^ but the supply is intermittent due to high demand on water infrastructure.^56^

There is no operational public sewage treatment facility in Accra, and at the time of the study, most fecal sludge from public and private toilets was discharged directly into the ocean by tanker trucks and marine outfalls. Coastal communities use beaches for economic (e.g., fishing and markets) and recreational purposes. Open drains transport excreta, wastewater, storm water, and solid waste throughout the city. A 2008 report described that over 50% of households discharge wastewater into open drains, and just under 10% of households safely dispose of wastewater in septic tanks.^57^ Average rainfall per year is 730 mm and occurs mostly during the two rainy seasons, May to mid- July and mid-August to October.^55^ Heavy rainfall and limited drainage infrastructure result in perennial floods, causing property damage and possible disease.^58,59^ Drain water containing human excreta is commonly used for irrigation of urban agriculture, which poses risks for farmers via contact, as well as risks to consumers through raw vegetable consumption.^39^

Overall, Accra presents diverse fecal exposure pathways that are representative of many sub-Saharan African cities and other cities in low-income countries. The setting afforded the opportunity to collaborate with strong in-country research partners as part of a multidisciplinary team. The research team included one of the top reference laboratories in sub-Saharan Africa, The Noguchi Memorial Institute for Medical Research of the University of Ghana (NMIMR); as well as the Water Research Institute (WRI) of the Council for Scientific and Industrial Research; the Training, Research, and Networking for Development (TREND) Group; and the International Water Management Institute (IWMI).

### Advisory boards.

Two advisory boards were convened to incorporate expert opinion into study design, methodology, and dissemination of results. One advisory board was comprised of ten international experts in the fields of environmental microbiology, behavioral data, QMRA, urban sanitation, and spatial data analysis. The other advisory board was comprised of fourteen Ghanaian leaders from government, academia, nongovernmental organizations (NGOs), and civil society. The international board provided input on methodological approaches, laboratory protocols, and international dissemination of results. The local board helped to identify pertinent fecal exposure pathways, advised on study neighborhood selection, helped contextualize findings, and guided dissemination of results within Ghana.

### Study neighborhood selection.

**Figure 2.**
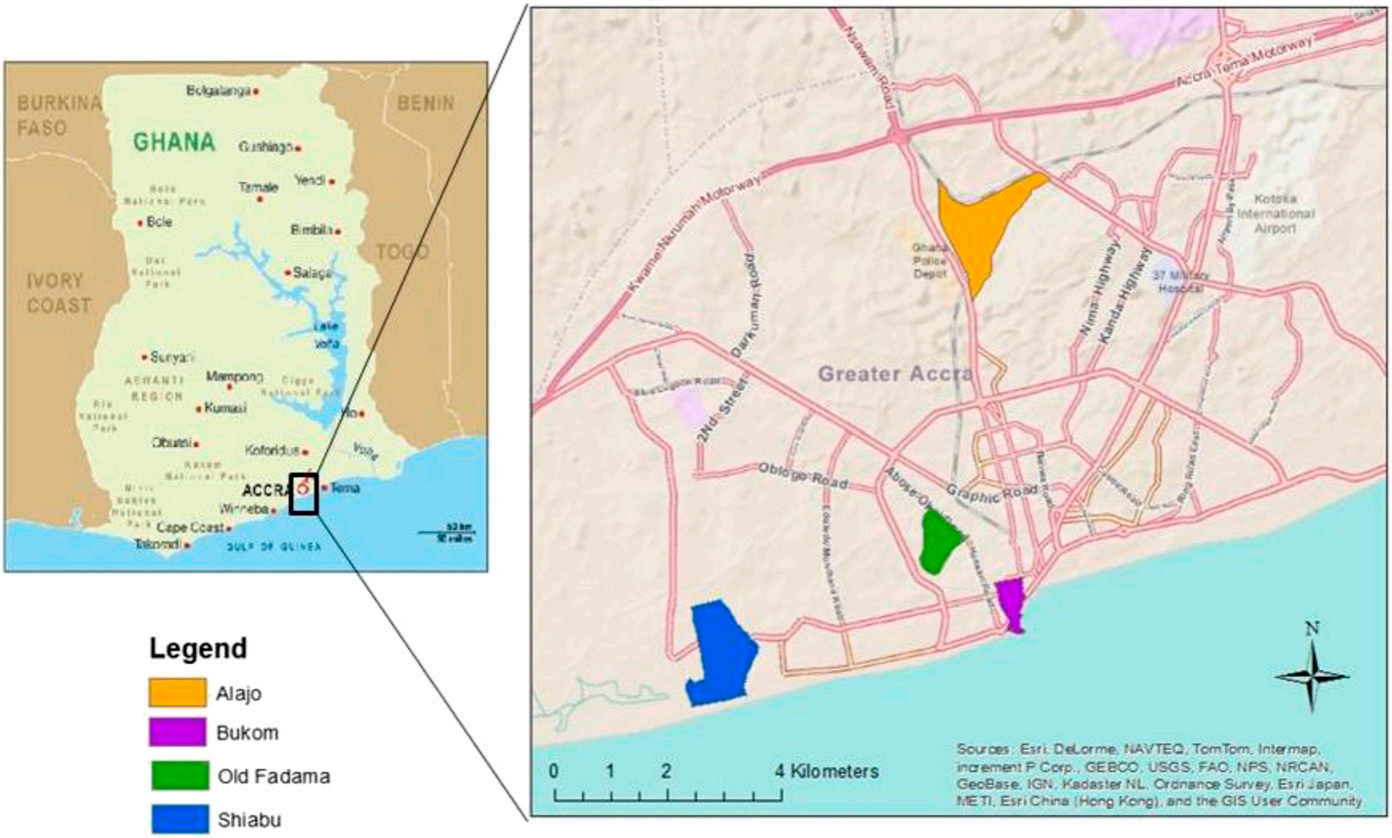
Study area: four low-income neighborhoods in Accra, Ghana. This figure appears in color at www.ajtmh.org.

Study neighborhoods were chosen to be representative of key risk conditions associated with poor containment, emptying, transport, and treatment of human excreta and also include a mix of socioeconomic status and religion. In addition to the local advisory board, informants from government, academia, and NGOs in Accra were consulted regarding neighborhood selection. Through this process, eleven candidate neighborhoods were initially identified. Key informant interviews, community mapping exercises, and focus group discussions were conducted to collect information on neighborhood demographics, physical characteristics, and feasibility of data collection. Four final neighborhoods were selected: Alajo, Bukom, Old Fadama, and Shiabu (Figure 2).

### Human subjects data.

Data on human subjects, including behavioral and demographic data, were collected to 1) characterize the study neighborhoods, and 2) identify and quantify the frequency and duration of behaviors that may involve contact with fecal contamination. The study protocol was approved by the Institutional Review Board at Emory University, GA (Protocol number: IRB00051584) and the University of Ghana Noguchi Memorial Institute for Medical Research Institutional Review Board (Protocol number: IRB00001276). Human subjects data were collected between June 2011 and September 2012. The study was verbally explained to each participant in the language they understood and each was given an opportunity to ask questions and have their concerns addressed. After voluntary agreement to participate, participants were consented. Literate participants were given written consent forms to read and sign. Illiterate participants were asked to provide a literate witness who signed on their behalf before they were asked, in the presence of their witness, to thumbprint the consent form. Human subjects data collection included focus group discussions (FGDs), key informant interviews (KIIs) with community leaders accompanied by neighborhood walks, household surveys, and structured observations of adults and children. The protocols are available on the study website (http://sanipath.org/the-tool/archived-protocols/), and the details are described in specific papers identified below.

#### Focus group discussions.

Two FGDs were conducted in each of six neighborhoods, including what would become the four study neighborhoods. One FGD type focused on neighborhood characteristics and residents’ daily routines. The other involved discussion of water, sanitation and hygiene practices. Detailed methods for the FGDs are described by Hurd and others.^60^ FGDs were conducted for multiple purposes: 1) to inform the neighborhood selection process, 2) as formative research for the design of household surveys, structured observation protocols, and selection of relevant fecal exposure pathways, and 3) to identify rare or sensitive exposures that may not be captured using quantitative methods for data collection, such as surveys or structured observations.

#### Key-informant interviews (KIIs) and neighborhood walks.

Eleven key informants were conducted with community leaders and Environmental Health Assistants (district officers responsible for neighborhood-level supervision of sanitation, waste management, food hygiene, etc.) regarding neighborhood characteristics and sanitation problems. After the KIIs, respondents were asked to take the study staff on a tour of the neighborhood to identify critical locations of exposure to fecal contamination (“hotspots”).

#### Structured observations.

Structured observations were conducted by trained observers to record the fecal exposure and mitigation behaviors of adults and children within the study neighborhoods. Enumerators recorded how adults and children came in contact with sources of contamination, what types of contact, for how long, and how frequently. Structured observations of the behaviors of children occurred in households, nurseries, primary schools, beaches, open drains, and public toilets. Structured observations of adults were conducted at beaches, open drains, markets, urban agricultural sites, and public toilets. Some structured observations data of adult caregivers in households were also collected. The structured observations not only provided critical information on exposure behavior but were also used to guide the collection of relevant environmental samples.

Structured observations were conducted for an average of 4.5 hours in 127 households, 22 nurseries, and 25 primary schools. Over 500 hours of structured observation of 129 children under age 5 years were conducted within households. In a subset of households, during the final hour of the observation, environmental samples (*N* = 441) such as food, hand rinses, water, and swabs of frequently touched surfaces were collected for microbial analyses. Environmental sample collection was guided by where and how exposure was observed to occur. The methods and results of the structured observations of children under 5 years of age in the household environment are described in Teunis and others.^61^

Eleven 4-hour observations (6 am–10 am) were conducted on beaches in the two coastal neighborhoods. Enumerators observed the frequency of open defecation and contact with sand and food, as well as the frequency and duration of water activities (swimming, wading). The locations where children swam and played were noted for collection of seawater and sand samples for microbial analyses.

Weekly observations at public toilets were conducted in each neighborhood. Data on user demographics and handwashing behavior were recorded. Observations were also used to inform sampling locations for surface and object swabs, hand rinses, and soil samples. The methods and results of the public toilet substudy have been reported by Peprah and others.^62^

Structured observations occurred at both urban agricultural sites and public markets where wastewater-irrigated produce was sold. Structured observations informed the location for soil, produce, drain water, fly, and water samples. The data collected at urban agricultural sites contributed to a risk assessment substudy on the use of wastewater in agriculture described by Antwi-Agyei and others.^63^

Forty-five, 1-hour structured observations of children and adults’ behavior around open drains were conducted in the study neighborhoods. The frequencies of contact with drains and type of contact (defecating into drain, entering drain, etc.) were recorded. Structured observations informed the locations for drain water sample collection. Data from this substudy contributed to an assessment of exposure to fecal contamination from open drains described by Gretsch and others.^64^

#### Household surveys.

To better understand the characteristics of the four study neighborhoods and assess the generalizability of the findings, 800 surveys (200 per study neighborhood) were conducted in a representative sample of households. Data were collected on population density, demographic characteristics, socioeconomic status, dwelling construction materials, extent of flooding, household sanitation infrastructure, WASH practices, and solid waste management.

### Environmental microbiology data.

The goal of the environmental sample collection was to characterize the order of magnitude and the variability in fecal contamination in all the studied environments and sample types. We originally planned to collect 10 samples of each type in each setting for each of the four study neighborhoods. In addition, for some types of samples we planned replicate sample collection during the dry season and the rainy season. Over 1,800 environmental samples of drinking water, ocean water, flood water, drain water, fecal sludge from public toilets, hand rinses, sand, soil, drain sediment, raw produce, vendor foods, surface and object swabs, and flies were collected and analyzed. Due to financial and logistical constraints, the realized sample numbers were different from the original plan. More samples were taken of surface waters (flood, ditch, sewage), drinking water sources, raw produce, and soil samples. Fewer samples were taken of sachet water, ocean water, vended food, hand rinse, sand, and sediment samples.

Ultrafiltration and membrane filtration methods were used to concentrate samples and *Escherichia coli*, enterococci, and coliphage were detected by standard culture methods; enteric viruses (norovirus and adenovirus) were detected by quantitative real time polymerase chain reaction. Details of the sample processing and microbiological analytic methods are provided on the study website (http://sanipath.org/the-tool/archived-protocols/).

#### Microbial target organisms.

The environmental samples were analyzed for microorganisms chosen to provide information on presence of overall and human-specific fecal contamination. The goal was to select enteric pathogens that were endemic in the local population, human-specific when possible, excreted by both adults and children, persistent in the environment, and not highly seasonal. In addition, the selection of target organisms considered whether there were quantitative laboratory methods to detect the organism in environmental samples because the exposure assessment models required quantitative data. Based on these criteria, some microorganisms were excluded. *Cryptosporidium parvum* and *Giardia lamblia* were not selected because detection of these organisms in environmental samples is not specific for human fecal contamination. *Campylobacter*, *Salmonella*, *Shigella*, and *Vibrio cholerae* were also excluded because they are less persistent in the environment than viruses and are more expensive to detect than *E. coli*. Furthermore, because detecting these pathogens in environmental samples usually requires an enrichment step, it is difficult to estimate the original concentration in the sample. Rotavirus was excluded due to the initiation of a vaccination campaign in Accra in 2012 and seasonal peaks in rotavirus infection in Ghana.^65,66^ The data from this study are some of the first, in addition to the work by Silverman and others, to establish the occurrence and concentration of enteric viruses in environmental samples in low-income urban environments.^67^

#### *Escherichia coli* and enterococci.

While *E. coli* and enterococci have limitations as surrogates for all enteric pathogens, they are commonly used as fecal indicator organisms for environmental samples and serve as markers of both human and animal fecal contamination. All environmental samples were analyzed for *E. coli*. Enterococci are more fecal-specific and persistent in the environment and have been used as indicators of hand hygiene.^68–70^ Hand rinse samples were analyzed for both enterococci and *E. coli* to assess both long-term and more recent fecal contamination.

#### Somatic coliphage.

Coliphage, viruses that infect *E. coli,* were selected as they are surrogates for enteric viral pathogens. They are more persistent in the environment and more resistant to water and wastewater treatment processes than fecal indicator bacteria.^71^ The detection of coliphage is easier and more economical than detection of human enteric viruses using PCR, making this a more feasible method for laboratories with limited resources.

#### Norovirus.

Noroviruses are epidemiologically important pathogens and may serve as an indicator of human fecal contamination in the environment.^72^ Infection with noroviruses is a common cause of epidemic and endemic viral gastroenteritis worldwide, in both adults and children.^73^ A study by Armah and others found that 15.9% of 89 diarrheal stool samples from Ghanaian children were positive for noroviruses, confirming the presence of norovirus in Ghana.^74^ Furthermore, noroviruses are very persistent in the environment and can indicate historic fecal contamination.^72,75^

#### Adenovirus.

Adenovirus serotypes 40 and 41 cause acute gastroenteritis and are a common cause of diarrhea in children worldwide. Several studies in sub-Saharan Africa report a high prevalence of adenovirus in pediatric stool samples and the detection of adenoviruses in various types of environmental samples.^76–78^ These viruses are relatively persistent in various environmental media, and infections may occur year-round.^79^ Adenoviruses 40 and 41 may be used as markers of human fecal contamination.^79^

### Data analyses.

The primary aim of this study was to conduct a quantitative assessment of exposure to fecal contamination for residents of low-income urban neighborhoods. Novel analyses of the behavioral data characterized the frequency, duration, and sequence of behaviors associated with exposure to fecal contamination. Behaviors that mitigated exposure were also included in the analyses. The structured observation data on children in households and nurseries were examined to quantify the probability that children exhibit key exposure behaviors (such as contact with contaminated floors or open drains). Network approaches were used to identify key sequences of activities (such as handwashing before eating, or bathing after defecation), and a quantitative behavior model was developed using behaviors and sequences of activities as determinants for contact with fecal microbes.^61^

Analyses of the environmental microbiology data examined the magnitude and distribution of the various target organisms in different sample types and within different exposure pathways. Based on rainfall data obtained from the Ghana Meteorological Agency, the environmental samples were classified by whether they were collected on either a day of and day following rainfall or a day without rainfall.

The analytic strategy for the exposure assessment combined quantitative data on behaviors that bring people into contact with fecal contamination and quantitative data on the magnitude of fecal contamination across the studied pathways. A novel simulation model was developed based on the analysis of child behavior in the private domain (Wang and others, companion paper).^80^ The model generated a timeline of child behaviors and corresponding locations where these behaviors occurred. Using the simulation model, a simulated child’s intake of microbes was estimated for all the studied exposure pathways within the household environment. For any behavioral state involving contact with the environment, the behavior and location were used to link to the appropriate environmental sample type and corresponding microbial concentration. For example, the behavioral event of a child playing on a dirt floor was linked with the concentration of fecal microbes in soil samples from within households. The timeline structure of the model followed a simulated child’s exposure to fecal contamination throughout an entire day, so that it was possible to infer the variability in the numbers of fecal microbes that attached and detached from the hand through repeated touching, and the numbers of fecal microbes that were ingested. The simulation model traced any microbes that were ingested via hands, food, water or soil back to their original source (i.e., soil, food, floors, open drains, surfaces, water, etc.) so that the contributions of each fecal exposure pathway could be assessed (Wang and others, companion paper).^80^

## KEY FINDINGS

Results of the SaniPath Study are presented in a series of articles and others that are in preparation.^60–64^ This section highlights and synthesizes the key study findings and summarizes the quantitative assessment of exposure to fecal contamination for young children within the household substudy. A detailed description of this analysis and results by Wang and others can be found in a companion article.^80^ Future manuscripts will describe the quantitative assessment of exposure to fecal contamination in the public domain for both adults and children.

The study neighborhoods are representative of a range of conditions characteristic of low-income urban neighborhoods in Accra (Table 1). Compared with the other three neighborhoods, Alajo has more residents of mixed socioeconomic status (SES), urban agriculture sites, private toilets, and large open drains. Bukom is an indigenous community with long-term residents and is located on the coastline with a beach used for economic activity (fishing, market, etc.). Bukom has a high prevalence of public latrine use. Shiabu is of mixed SES, close to a citywide fecal sludge dumping site (Lavender Hill), and has a beach with much less public activity than in Bukom. Old Fadama is considered an illegal squatter settlement by the government and thus has very little infrastructure, is home to new immigrants to Accra, and is comprised of a very low SES population. Over half of all compounds in each neighborhood operated a business from their home. Across the four neighborhoods, the proportion of households with sanitation ranged from 2% to 58%, and there was heavy reliance on shared toilets and public pay-per-use toilets. Over 60% of 400 households surveyed in two of the study neighborhoods reported using public toilets every day despite dissatisfaction with the cleanliness, service and costs of public toilets.^62^ While many key characteristics of the neighborhoods of Alajo and Shiabu are similar (e.g., similar population density and proportion with in home/compound sanitation), the average estimated daily exposure dose of fecal contamination for young children was more than 10,000 times higher in Shiabu compared with Alajo^80^ – suggesting that exposure to fecal contamination is determined by more complex factors than population density and sanitation coverage.

**Table 1:** Characteristics of study neighborhoods

Population characteristic	Neighborhood			
	Alajo	Bukom	Old Fadama	Shiabu
Population density* (per square km)	21,475	75,927	50,835	21,594
Estimated total population*	34,360	27,030	28,010	32,520
Neighborhood area (square km)	1.60	0.36	0.55	1.51
Type of settlement	Formal	Formal	Squatter	Mixed
Location	Inland	Coastal	Inland	Coastal
Flood prone	Yes	No	Yes	Yes
Near major market	No	Yes	Yes	No
Data below based on survey of 800 households (200 per neighborhood)				
% With child under 5	42	46	55	45
% With no formal education	13	14	44	9
% Christian	79	88	38	97
% Muslim	22	8	61	3
% Own their home	52	80	64	55
Average years of residency	14	28	8	12
Average no. households in compound	7	5	2	9
% Operating business from compound/home	60	68	51	57
% Keeping animals in the compound/home	65	42	28	70
% Using sachet water as primary drinking water source	77	72	93	75
% With sanitation facility in compound/home	58	7	2	46
% With refrigerator in the compound/home	74	53	30	73
% Of compounds/homes that own a car	26	6	4	17

* Ghana Statistical Service (2013) 2010 Population and Housing Census, Accra, Ghana.^48^

Over 1,800 environmental samples were collected and analyzed for *E. coli*, enterococci, coliphage, norovirus, and adenovirus (Table 2). Many of the sample types had highly variable concentrations of fecal microorganisms; therefore, the total number of samples collected of each sample type was influenced, in part, by the observed variability in concentrations of fecal microorganisms (i.e., more samples were collected from sources with highly variable concentrations). The majority of samples were concentrated in the household substudy. Sample types with the largest number of samples included hand rinses (*N* = 287), produce from markets (*N* = 204), soil samples (*N* = 273), and surface swabs (*N* = 273). Sample collection extended over 10 months, during both rainy and dry seasons, to examine the effect of seasonality on fecal contamination in the environment. Of the 1,845 environmental samples tested for *E. coli*, 31% were collected on a day of or a day after a rainfall event. Overall, the median log_10_ *E. coli* concentration in samples collected on days without rainfall was similar to that for samples collected on days with, and days immediately following, rainfall.

**Table 2:** Target organisms and substudies associated with each sample type

	Target organism						Substudy								
Sample type	Total sample number	*Escherichia coli*	Enterococci	Somatic coliphage	Norovirus (GI and GII)	Adenovirus 40 and 41	Household	Primary school	Nurseries	Urban agriculture	Markets	Public latrines	Open drains	Beaches	Flood zones
Hand rinses	287	X	X				X	X	X			X			
Municipal piped water	117	X		X	X	X	X	X	X						
Stored sachet water	61	X			X	X	X	X	X						
Other stored drinking water	63	X			X	X	X	X	X						
Ocean water	38	X		X	X	X								X	
Drain water	91	X		X	X	X							X		
Irrigation water	87	X		X	X	X				X					
Flood water	12	X			X	X									X
Produce on farm	87	X		X	X	X				X					
Produce in market	190	X		X	X	X					X				
Vendor food	38	X		X	X	X									
Sand	46	X		X	X	X								X	X
Soil	273	X		X	X	X	X	X	X	X		X			X
Sediment	57	X			X	X							X		
Flies	61	X			X	X	X				X	X	X		
Surface swabs	273	X			X	X	X	X	X			X			
Fecal sludge from public latrines	40	X		X	X	X						X			
Other	34	X		X	X	X	X	X	X						
Total sample number	1855	1845	285	314	1303	1461	434	157	138	256	216	226	160	76	42

“X” indicates that a sample type was tested for a target organism or associated with a particular substudy.

Overall, the results indicate widespread and often high levels of fecal contamination in these poor urban neighborhoods and frequent direct contact and exposure to feces by adults and children. Sanitation access and services, as well as fecal sludge management, were generally poor. About 20% of household toilets discharged directly into open drains that were nearly ubiquitous along every street and alley. Spatial analyses are in progress to examine the distribution of fecal contamination throughout the neighborhoods.

The results from the focus group discussions indicated that the residents were very aware of fecal contamination in the public domain and frustrated at how difficult it was to avoid exposure to this contamination, both for themselves and for their children, during their range of daily activities^60^ The focus group participants also described numerous ways that fecal contamination in the public domain entered their households via feet and shoes, flooding of clogged open drains, and wind and rain moving fecal-contaminated soil and refuse into the household. This concern is substantiated by our microbiological analyses of household samples indicating high concentrations of *E. coli* in soil (geometric mean *E. coli* concentration [CFU per gram] 460.4, range: 0.9–8,000) and on indoor surfaces (surface swab geometric mean *E. coli* concentration [CFU per cm^2^], 3.5 range: 0.1–98.4).

In the household domain, data from structured observations were quantitatively modeled to describe child behavior in the four study neighborhoods over the course of a typical day.^61^ Very young children (< 1 year) spent most of their time playing or sleeping off the ground, but children between the ages of 1–5 years frequently played and ate while sitting on concrete or dirt floors of the household. The structured observations also documented that handwashing rarely occurred after defecation or before eating.^61^ Microbiological analyses of hand rinse samples from young children showed the presence of high concentrations of *E. coli* and enterococci (geometric mean *E. coli* concentration [colony-forming units (CFU) per pair of hands] = 247.9, range 2.3–3.2 × 10^4^; geometric mean enterococci concentration [CFU per pair of hands] = 1,941, range: 22.5–8.4 × 10^4^).

The QMRA focused on exposures of young children in the private domain because that is where they spent the majority of their time. Estimated total fecal exposure via ingestion varied by child age and neighborhood and ranged between 10^8^–10^16^ CFU per day of *E. coli* (Wang and others, companion paper).^80^ Contaminated food contributed more than 99.9% to the total fecal exposure for children under five in all study neighborhoods. Microbiological results indicated that the produce, which was usually irrigated with wastewater from the open drains, was highly contaminated (geometric mean *E. coli* concentration [CFU per produce item] = 5,910.8, range 1.1–10^6^). High proportions of both adults and children reported frequently eating uncooked vegetables. Hands played a pivotal role in the transfer of fecal microbes from contaminated environmental surfaces to ingestion. The municipal water supply contributed only a small portion of fecal exposure because of the lower levels of *E. coli* and low frequency of reported consumption. Across the four study neighborhoods, 80% of residents reported sachet water as their primary drinking water source. Sachet water consumption was especially prevalent in the poorest neighborhood, Old Fadama. Quantitative assessment of the risk associated with contact with open drains for children under 13 years of age (based on structured observations and microbiological analyses) demonstrated that any contact was associated with a high exposure to fecal microbes because of the magnitude of contamination (*E. coli* geometric mean = 4.5 × 10^8^ CFU/100 mL, range = 4.5 × 10^6^–1.5 × 10^11^ of drain water).^64^

Future manuscripts will describe and compare exposure to fecal contamination across pathways in the public domain for both adults and children.

## IMPLICATIONS

Previous studies of Accra have concluded that the open drains posed the greatest risk to public health.^50^ The SaniPath Study also observed substantial risk associated with any contact with open drains because of the magnitude of fecal contamination in these drains.^64^ However, for young children, our results indicate that the dominant exposure pathway was through food and demonstrate the critical link between poor sanitation and food safety. This has important implications for the “WASH sector” – that typically includes only water, sanitation, and hygiene and ignores food safety. Urban agriculture is a key contributor to the food supply in many cities in sub-Saharan Africa, and wastewater irrigation is a common practice.^39^ Yet, our study reveals that this exposure pathway combines high frequency of exposure and high “doses” of fecal contamination – making it a high-risk pathway that should be a priority for intervention (Wang and others, companion paper). Furthermore, produce can be a vehicle for fecal contamination to move across the city from poor urban neighborhoods into middle- upper-income neighborhoods due to the fact that markets may sell produce grown in these neighborhoods all over the city. Finally, the large proportion of adults and children that reported regularly consuming produce also points to a shift away from traditional diets among urban populations. This may be due in part to heavily reliance of poor populations on street-vended food with entrees that often included salads.^81^ Another shift in consumption habits is represented by the low rates of reported consumption of piped municipal water, a finding that was also observed by Stoler and others.^82^

This study also indicates the important interconnections between the different fecal exposure pathways. Poor sanitation and fecal sludge management leads to fecal contamination of open drains and the food supply. Widespread fecal contamination in the public domain is also reflected in high levels of *E. coli* on floors and surfaces in the private domain that may in turn cause further contamination of hands, food and objects that come into contact with these surfaces and then enter the mouths of young children. Furthermore, the spatial distribution of fecal contamination in the neighborhood can be influenced by clusters of sanitation infrastructure or behaviors. Because of these complex interactions, the risk of exposure to fecal contamination cannot be predicted by neighborhood-level estimates of population density and sanitation coverage and requires data on multiple exposure pathways and use of sophisticated modeling approaches.

In Ghana, in response to the study findings, the Ministry of Local Government and Rural Development, in conjunction with the Ministry of Water Resources, Works and Housing, set up a committee of experts to mainstream the study findings and recommendations into national communication campaigns against poor sanitation. Further in-country dissemination of study findings, with the support of the local advisory board, has occurred through policy briefs, neighborhood meetings, national conferences, and television interviews highlighting the study findings and public health risks of poor sanitation (see SaniPath.org).

The SaniPath Study started with a 16-month, in-depth, interdisciplinary exposure assessment to identify, characterize, and prioritize fecal exposure pathways. While it was a necessary first step to create a very detailed picture of the fecal exposures in a range of low-income, urban neighborhoods, this type of resource-intensive data collection process is not feasible for most low-income cities. This first phase of the SaniPath Study served as both as a proof-of-concept to demonstrate that a comprehensive fecal exposure risk assessment could be accomplished in a low-income country setting and also to identify what data and methods were critical for the exposure assessment.

This study had some limitations. First, we recognize that these results come from only four neighborhoods in a city of approximately 2 million people. Although these neighborhoods were chosen to represent a range of conditions in low-income neighborhoods, our findings may not be generalizable to all of Accra or other cities in sub-Saharan Africa. In particular, these findings may not be generalizable to cities in dry climates or where there is better fecal sludge management. Different cultural norms and practices will influence exposure behavior – such as the type and coverage of sanitation facilities, increased reliance on municipal drinking water, routine household water treatment, diets that do not include consumption of raw produce, or where wastewater is not used for irrigation of food crops.

Despite the large number of environmental samples that were collected in this study, it was still not possible to cover the geographic area of the whole neighborhood. Our sampling strategy was purposeful and was based on reported exposures and hazards. However, this resulted in some geographic locations with few or no samples. Some samples, such as fecal sludge from public latrines, proved to be extremely difficult to access and collect. When the environmental samples are stratified by type, neighborhood and season, the sample size becomes small and limits some types of analyses.

The microbiological data generated in this study make a significant contribution to the limited evidence base regarding environmental contamination of low-income, urban neighborhoods. The intent of the microbiological analyses was not to make high resolution comparisons between different types of samples or contamination in different neighborhoods, but rather to provide information on the order of magnitude of fecal contamination in different compartments of the environment and identify large differences between neighborhoods to help prioritize interventions. While this goal was met, we recognize that there are inherent limitations to the use of fecal indicator organisms and methods to detect pathogens in environmental samples. One important limitation for sanitation investment decisions is lack of differentiation between human and animal fecal contamination.

While we collected extensive structured observation data in households, our observation data in the public domain (beaches, schools, public latrines, nurseries, and open drains) is much less comprehensive, and this has been challenging for our QMRA analyses of exposure pathways in the public domain.

Finally, this QMRA study focuses on risks of exposure and it is difficult to extrapolate from exposure to health effects. However, health effects are conditional on exposure and more knowledge of exposure characteristics can guide the design of relevant interventions.

### Future directions.

Applying the lessons from the in-depth investigation in Accra, the second phase of the SaniPath Study developed a streamlined tool to compare risks of exposure to fecal contamination within and across neighborhoods of a city using lower-cost, lower-resource approaches. The SaniPath Tool (see SaniPath.org for more information) is a simplified, but still informative and rigorous, means to compare exposure risks using a basic assessment of the magnitude of environmental contamination (concentration of *E. coli*) and the frequency and types of exposure behaviors of adults and children. It considers risks of exposure to fecal contamination from multiple exposure pathways associated with inadequate sanitation and fecal sludge management infrastructure and services. The tool guides users in understanding which components drive risk (frequency of behavior versus magnitude of fecal contamination) and which pathways contribute the most to the risk of exposure to fecal contamination in specific contexts. Development, application, and evaluation of this tool in Accra, Ghana; Vellore, India; Maputo, Mozambique; Siem Reap, Cambodia; and Atlanta, United States have recently been completed and will be described in future reports. These applications of the tool will allow us to compare exposure behaviors and magnitude of fecal contamination in the public domain in diverse urban conditions.

The 2015 Sustainable Development Goals (SDG) include targets for not only improving access to toilets but also halving the proportion of wastewater that does not receive treatment. The fecal waste flow diagrams from many cities show that most fecal sludge stays in the residential environment and is untreated.^18^ Currently, poor sanitation infrastructure in many low-income, urban neighborhoods fails to prevent human contact with fecal contamination. Cities are particularly important places for identifying dominant fecal exposure pathways due to the size and density of the populations at risk, the complex nature of human-human and human-environment interactions, and the overlap of risks in the public and private domains. Innovations in sustainable sanitation services and policies in urban settings are contingent on better understanding of the nature of fecal exposures in urban environments and the associated public health risks. Comprehensive data on multiple exposure pathways in urban settings can be used to better target interventions toward the fecal pathways that pose the greatest exposure risk to vulnerable populations. The SaniPath Study is a first step to improve our understanding of risks from fecal contamination in urban settings in low-income countries and ultimately develop effective strategies to reduce these risks.
